# Short-term mTOR inhibition by rapamycin improves cardiac and endothelial function in older men: a proof-of concept pilot study

**DOI:** 10.1007/s11357-025-01855-8

**Published:** 2025-09-19

**Authors:** Alexander J. Moody, Yubo Wu, Terry Q. Romo, Wouter Koek, Jinqi Li, Leslie A. Linehan, Geoffrey D. Clarke, Eric Y. Yang, Sara E. Espinoza, Nicolas Musi, Robert J. Chilton, Ellen Kraig, Dean L. Kellogg

**Affiliations:** 1 Barshop Institute, University of Texas Health Science Center at San Antonio, San Antonio, TX USA; 2 Department of Radiology and Imaging Sciences, Indiana University School of Medicine, Indianapolis, IN USA; 3 Department of Rehabilitation Medicine, University of Texas Health Science Center at San Antonio, San Antonio, TX USA; 4 Department of Cell Systems and Anatomy, University of Texas Health Science Center at San Antonio, San Antonio, TX USA; 5 Research Imaging Institute, University of Texas Health Science Center at San Antonio, San Antonio, TX USA; 6 Department of Medicine, University of Texas Health Science Center at San Antonio, 7703 Floyd Curl Dr., San Antonio, TX USA; 7 Department of Medicine, Cedars-Sinai Medical Center, Los Angeles, CA USA; 8 GRECC, South Texas Veterans Health Care System, San Antonio, TX USA

**Keywords:** Rapamycin, mTOR, Cardiac MRI, Diastolic function, Endothelial function, Inflammation, Clinical trial

## Abstract

Rapamycin (RAPA) and related mTOR-inhibitors have been shown to enhance healthy aging in animal models (2–10) and to be generally safe and tolerable in older people (11–13). However, studies to assess their effects on specific age-related pathologies in humans are limited. Since improvements in cardiovascular function with RAPA treatment have been reported in preclinical studies (5, 9, 10, 14–17), we posited that RAPA could be similarly efficacious in humans. Towards this end, we performed a pilot “proof of concept” trial to examine RAPA’s effects on cardiovascular and endothelial functions that are known to decline with age (18, 19). We hypothesized that RAPA would elicit beneficial cardiovascular effects in men. A cohort of older male subjects with no known cardiac disease (ages 70–76 years) were enrolled in the open-label study and received 1 mg RAPA/day for 8 weeks. To assess cardiovascular function, cardiac MRI (CMR) was performed twice: prior to initiation of the intervention and again after 8 weeks of treatment. Endothelial function was examined using laser-Doppler flowmetry (LDF) by measuring cutaneous, endothelium-dependent, local thermal hyperemic responses pre-intervention and after 4 and 8 weeks of RAPA (1). In all 6 subjects, transmitral blood flow, peak flow rate, and maximal blood acceleration showed statistically significant improvements while endothelial function also improved over the 8-week course of RAPA treatment. Thus, cardiac and endothelial function improvements with RAPA were found and support future placebo-controlled studies in larger cohorts of healthy older persons as well as in patients with compromised diastolic and endothelial function (20, 21).

## Introduction

Inhibition of the mechanistic target of rapamycin (mTOR) pathway by rapamycin (RAPA, sirolimus) enhances longevity in mice even when treatment is initiated relatively late in life [22]. Concomitant improvements in age-related deficits in cognitive, immunological, and cardiovascular function have also been reported with RAPA treatment in animal models [5, 7, 8, 10, 23]. For example, an early investigation by Flynn, et al. [16], in 24-month-old mice treated with 14 ppm RAPA for 3 months, found that this treatment attenuated age-related cardiac hypertrophy and marginally improved systolic function; these changes were accompanied by reduced inflammatory cytokines in blood and even more so in cardiac tissue. Similarly, Dai, et al. [17] demonstrated that RAPA administration for 10 weeks improved diastolic function and reversed cardiac hypertrophy through attenuated age-dependent protein damage, decreased protein turnover rate, and enhanced proteome remodeling in 25-month-old female mice. Moreover, Quarles, et al. [14] examined RAPA cardiac effects in both male and female C57BL/6 mice and found improvement in diastolic function with an increase in LV compliance and reduced myocardial hypertrophy in both sexes with 8 wks of RAPA treatment which persisted for at least another 8 weeks after RAPA cessation. Subsequently, Urfer, et al. [10], examined cardiac function outcomes in middle-aged healthy dogs after 10 weeks of systemic RAPA or placebo and found that RAPA treatment significantly improved fractional shortening with attendant strong trends toward improvement of the E/A ratio [ratio of peak blood flow velocity in early diastole (E wave) to peak velocity flow in late diastole due to atrial contraction (A wave)], and left ventricular ejection fraction (LVEF).

The foregoing pre-clinical data suggest that RAPA might delay age-related cardiac functional deficits in humans, potentially improving healthspan. This premise is consistent with the geroscience hypothesis that targeting fundamental aging processes, like the mTOR system, might ameliorate such deficits. We previously showed that RAPA administered at an oral dose of 1 mg daily in elderly persons (age 70–95) for 8–16 weeks was relatively safe and generally well tolerated, with no diabetes related to treatment observed [24]. We subsequently began to seek evidence of beneficial effects of RAPA to reverse age-related condition(s) in the same demographic of elderly humans. Given the supportive pre-clinical data, we focused on cardiovascular effects since it is well documented that baseline systemic inflammation, which is increased in older persons, contributes to the development of age-related pathologies affecting both the heart and the vasculature [25–27]. Furthermore, evidence indicates that alterations in both inflammatory and pro-fibrotic pathways are critically implicated in age-related cardiovascular dysfunction [28] and are reported to be ameliorated by RAPA treatment in preclinical models [5]. These alterations lead to the development of reduced LV reserve capacity with attendant dysfunction manifested as compromised ability to respond to stress. For example, LV diastolic function declines with advancing age such that older persons have less complete cardiac relaxation; this leads to compromised cardiac output as compared to younger individuals. In particular, stiffening (decreased compliance) in the aged LV myocardium contributes to diastolic dysfunction without apparent effect on ejection fraction (EF); thus, age is an important predictor of the development of heart failure with preserved ejection fraction (HFpEF) [29].

In humans, LV hypertrophy and increased LV myocardial mass frequently occur after heart transplantation and ultimately compromise LV function. Raichlin et al. [30], investigated the effects of 12 months of RAPA as the primary immunosuppressant on LV hypertrophy and diastolic dysfunction in 70 cardiac allograft recipients. Outcomes were monitored by echocardiography. Enrolled patients had been treated with calcineurin-inhibitors (CNI) for primary immunosuppression for 5.8 ± 3.9 years and were withdrawn from all CNI agents and converted to RAPA. One year after conversion, patients had significant reductions in LV myocardial mass and left atrial volume, markers of improved diastolic function. Overall, these results suggest that RAPA is capable of improving aspects of cardiac function, particularly LV diastolic function which tends to decline with age in humans.

In addition to cardiac manifestations, both the macro- and microvasculature systems are altered by aging in humans [28]. These age-related changes are etiologically linked to endothelial dysfunction including reduced nitric oxide (NO)-dependent vasodilation [31, 32]. Important pre-clinical work in B6D2F1 hybrid mice by Lesniewski et al. [5] showed that microvascular dysfunction was improved with only 6 weeks of RAPA treatment in old mice [30.3 ± 0.6 mo. (mean ± SEM), an age corresponding to an ~ 80–83 year old human [33–36]] through improved NO-mediated, endothelium-dependent vasodilation, likely mediated by decreased vascular oxidative stress and attenuated quenching of NO by reactive oxygen species (ROS). Interestingly, no such effect was detectable in younger animals [3.8 ± 0.6 mo. (mean ± SEM)] treated with the same dose of RAPA. These results suggest that mTOR produces increased vascular oxidative stress with consequent NO quenching and reduced microvascular NO bioavailability. However, these outcomes only become evident once deficits due to aging are present. In addition, they reported that collagen content in the aorta was reduced by RAPA, consistent with regression of cardiovascular fibrosis. The findings of Lesniewski et al. [5] that RAPA reversed age-associated endothelial dysfunction and macrovascular fibrosis suggest that RAPA could have similar beneficial effects in older human subjects.

Therefore, we posited that RAPA treatment would produce improvements in cardiac and endothelial function in older humans. In order to test this hypothesis, we administered RAPA (1 mg/day) orally for 8 weeks to 6 male septuagenarians and examined cardiac outcomes using cardiac MRI (CMR) and endothelial effects by laser-Doppler flowmetry (LDF) measurements of cutaneous vascular thermal hyperemic responses; the latter are mediated primarily by NO production by the endothelium [1, 37]. Measurements were obtained before, during, and after open-label RAPA treatment in a small proof of concept study [18, 19].

## Materials and methods

### Ethical approval

The study was approved by the Institutional Review Board at the University of Texas Health Science Center at San Antonio (UTHSA) and complied with the Declaration of Helsinki. The study was also monitored by the UTHSA Claude D. Pepper Data and Safety Monitoring Board and was registered as NCT02874924 at www.clinicaltrials.gov.

### Protocol design

We performed an open label, pilot “pre-post” clinical trial as a “proof of concept” test of whether short term RAPA treatment was safe, tolerable, and possibly improved cardiac and/or endothelial function. Clinical aspects of the project were performed at UT Health-San Antonio and at the South Texas Veterans Health Care System Frederic C. Bartter General Clinical Research Center. Informed consent was obtained from all participants.

### Participants

Subjects enrolled in this study were community-dwelling, septuagenarian men in good health with all medical problems clinically controlled and stable. None had active cardiac disease, none were being treated for hypertension, and none had ever taken RAPA or participated in our prior RAPA trial [24]. Exclusion criteria included: prior history of cardiovascular conditions, arrythmias, heart failure, or abnormal ECGs, smoking, diabetes, cancer, use of systemic immunosuppressants (steroids, etc.) within the prior year, or any history of poor wound healing. Additionally, subjects were excluded if they received medications known to interact with RAPA or directly affect cardiovascular function; statin use was permitted. CMR exclusion criteria included the presence of metallic foreign bodies, MR unsafe and conditional implanted devices or materials, and mental or auditory impairments which would have precluded them from complying with operator commands or remaining still in a closed space for extended periods of time.

### Intervention and protocol timeline

All subjects received oral doses of RAPA (sirolimus) of 1.0 mg per day over a course of 8 weeks. No placebo or control group was involved in the current pilot “proof of concept” trial. Safety lab and local thermal hyperemia data were collected prior to initiation of RAPA treatment, after 4 weeks of treatment, and after 8 weeks RAPA. The final local thermal hyperemia measurement was made 48 h after the last RAPA tablet was ingested. Cardiac MRI images were acquired at two time points, once before and once following 8 weeks of daily RAPA. The final CMR was done 24 h after the last RAPA tablet was ingested. During the course of treatment subjects were asked not to significantly alter their lifestyles, diets, or physical activity. The timeline of the protocol is delineated in Table 1.

**Table 1:** Protocol timeline

Visit goal	Consent/screen	Pre-RAPA	1 wk RAPA	4 wks RAPA	8 wks RAPA
WEEK NUMBER		0	1	4	8
Consent	●				
Geriatric depression scale	●				
CLOX>10	●				
History and Physical	●	●	●	●	●
SAFETY/AEs/TESTS					
Self-reported Adverse Events			●	●	●
CBC and CMP	●	●			●
FBS/lipids,	●	●			●
HbA1c	●	●			●
Inflammatory Mediators		●			●
ECG	●	●			●
Rapamycin level			●		
CARDIOVASCULAR FUNCTION					
Cardiac MRI		●			●
Cutaneous Thermal Hyperemia		●		●	●
PHYSICAL FUNCTION					
Hand grip and 40-foot walk		●		●	●

Clinical tests: Complete blood count (CBC); Complete Metabolic profile (CHEM-20 = sodium (Na), potassium (K), chloride (Cl), CO_2_ (bicarbonate), creatinine, glucose, urea nitrogen (BUN), albumin, calcium, inorganic phosphorus, alkaline phosphatase, ALT/SGPT, AST/SGOT, total bilirubin, total protein, creatine kinase); Fasting blood sugar (FBS) and hemoglobin A1c levels; Fasting lipid profiles (FLP = total cholesterol (TC), high density lipoprotein (HDL), low density lipoprotein (LDL), triglycerides (TG), very low density lipoprotein (VLDL))

### Clinical assessments

The following procedures and tests were performed prior to initiation of RAPA treatment and also at 4 and 8 weeks: i) Standard history, vital signs, and physical examinations as well as 12-lead EKGs to assess general health and to detect subsequent adverse reactions and subclinical sequelae of participation; ii) Complete blood count (CBC); iii) Metabolic profile (CHEM-20 = sodium (Na), potassium (K), chloride (Cl), CO_2_ (bicarbonate), creatinine, glucose, urea nitrogen (BUN), albumin, calcium, inorganic phosphorus, alkaline phosphatase, ALT/SGPT, AST/SGOT, total bilirubin, total protein, creatine kinase): iv) Fasting blood sugar (FBS) and hemoglobin A1c levels; v) Fasting lipid profile (FLP = total cholesterol (TC), high density lipoprotein (HDL), low density lipoprotein (LDL), triglycerides (TG), very low density lipoprotein (VLDL)). Blood RAPA levels were ascertained by mass spectroscopy performed by the UTHSA Biological Psychiatry Analytical Lab [24, 38].

### Physical performance measures

To examine functional sequelae of RAPA administration, physical performance measures were done in identical fashion longitudinally to offer the greatest potential to detect changes and included: i) Handgrip (HG) strength done while seated in a chair with the forearm at a 90° elbow bend with 3 consecutive grip strengths completed with a standard grip strength dynamometer for both hands; and ii) Timed walks were performed by each participant at their preferred walking speed over a measured 40-foot path.

### Cardiac MRI (CMR)

CMR was chosen as our imaging modality due to the excellent reproducibility and superior contrast resolution in cardiac tissues; all CMR images were acquired on a Siemens 3 T TIM Trio MRI system (Siemens Healthineers, Malvern, PA). Subjects were connected to an ECG to monitor vital signs and for cardiac gating. The MR operator provided instructions on breath holding via an intercom system.

Cine images were acquired in 3 orthogonal image planes: a short-axis view with slices spanning all four heart chambers (SAX), a long-axis (LAX) 2-chamber view of the left atrium and ventricle (2CV), and a LAX 4-chamber view (4CV). A retrospectively gated, balanced steady state free-precession pulse sequence was used to acquire each of the three image sets (TE = 1.22 ms, TR = 2.44 ms, 25 frames). Images were acquired by slice and retrospectively reconstructed according to the ECG signal at the time of acquisition. In total, data were arranged in 25 frames from end-diastole to end-diastole for each heartbeat.

Through-plane 2D phase-contrast cine images were taken parallel to the mitral valve plane at the interface of the left atrium with the LV. A velocity-encoded preparatory cine sequence with a simultaneous bright-blood, T1-weighted magnitude acquisition (TE = 84.5 ms, TR = 3.86 ms, flip angle = 15°, voxel size = 3.0 × 2.2 × 8.0 mm, 25 frames) was used to obtain mitral flow images. Flow images were retrospectively reconstructed according to ECG signal and paired with the normal cine images. Typically, 25 frames from end-diastole to end-diastole over one heart cycle were acquired.

### CMR image analysis

All images were analyzed using cvi42^©^ cardiac image analysis program (v5.14.2; Circle Cardiovascular Inc, Calgary, AB, Canada). A standard feature-tracking approach was utilized to measure volumetric endpoints in the LV [39]. This analysis involved contouring the systolic and diastolic frames in all three image views and slices where the LV was visible. Contours were drawn to annotate the endocardial and epicardial boundaries of the LV. In the two ventricular systolic and diastolic long axis (LAX) views, a T-shaped measurement tool was used to identify the mitral valve plane and the LV central axis length. All contours were combined in a 4D space to produce a wire-frame model of the LV. The total volume of the LV was measured at end-diastole and end-systole to derive functional cardiac parameters. These parameters were used to calculate clinical metrics of diastolic and systolic function.

Mitral flow images were measured using a pixel-based region of interest (ROI) approach. In all frames of the magnitude cine acquisitions, the mitral valve was annotated with a ROI. Any pixels residing within the aortic lumen were excluded from this ROI while maximizing the number of voxels in the mitral valve. Background and static tissue corrections were applied to each image set to exclude artifactual data. Anti-aliasing corrections were applied to compensate for velocity measurements outside of the acquisition range. Pixel values within the ROI were correlated with velocities to derive the minimum, maximum and mean values of flow rates and velocities. Acceleration was obtained from the time derivative of velocity, and transmitral blood volume was calculated from the integral sum of flow rate over time. Data are reported as either a maximum or peak of the quantity indicating the largest pixel quantity and the mean of all pixels within the ROI, respectively.

### Endothelial function

To assess the effect of RAPA on NO-mediated, endothelium-dependent vasodilation in the microvasculature, we measured local thermal hyperemic responses in the skin before RAPA was started, after 4 weeks of RAPA, and after 8 weeks of RAPA treatment in all participants. The protocol used was originally developed by Choi et al. [1]. They used LDF to measure hyperemic skin blood flow responses during gradual local heating to 39 °C combined with intradermal microdialysis at four forearm skin sites treated with: 1) lactated Ringer solution (Control); 2) tetraethylammonium (TEA) to inhibit EDHFs; 3) nitro-L-arginine methyl ester (L-NAME) to inhibit NO synthase; and 4) combined TEA and L-NAME. They found that the vast majority of the thermal hyperemic response was mediated by NO, based on the effects of the different antagonists. They therefore proposed that local heating to 39 °C represented a novel and safe approach for determining NO-dependent dilation and/or assessing perturbations that may improve microvascular function. This approach has proven to be the most robust test of NO-dependent dilation that can be performed non-invasively in humans, as the multifold increase in conductance from 34 to 39 °C is almost entirely mediated by NO generation [1]; we thus used the local thermal hyperemia approach in our study.

Upon arrival in the laboratory, subjects were placed in a semi-supine position in a dialysis chair and instrumented to measure LDF from forearm skin. The LDF probe was equipped with a heating element and thermocouple to permit simultaneous LDF measurements and control of local skin temperature. After a 5-min normothermic baseline period, local skin temperature at the LDF measurement site was rapidly heated to 39 °C and held at that temperature. The LDF response was recorded for approximately 30 min after which local skin temperature was increased to 42 °C and held at that temperature for 25–40 min to evoke maximal vasodilation for data normalization [1, 40, 41]. Absolute values of LDF (in mV) with local skin temperature held at 39 °C were ascertained and normalized to maximal LDF levels at 42 °C for analysis.

### Inflammatory mediators

One 8.5-ml serum tube of blood (black/red tiger top; BD367988, Becton Dickinson) was collected from each subject, mixed by inversion, and then allowed to clot at room temperature for 30’. The tubes were spun for 10’ at 1100 × g, and the serum was then frozen in 0.7 ml aliquots and stored at −20 °C. Serum samples were thawed and spun at 10,000 g for 10 min before use in ELISAs. Commercial ELISA kits were used to measure levels of inflammatory mediators; these included: s(soluble)ICAM-1 [Invitrogen/ThermoFisher kit # BMS201], RAGE [Invitrogen/ThermoFisher kit # EHMOK], and TGF-β [Invitrogen/ThermoFisher kit #BMS-2065]. These three kits were performed with a 1:10 serum dilution according to the manufacturer’s specifications. In addition, a high sensitivity IL-6 assay [Invitrogen/ThermoFisher kit # BMS213HS] was performed according to the manufacturer’s instructions with serum at a 1:2 dilution.

### Statistical analysis

Our primary hypothesis was that RAPA administration would be safe and tolerable. Our exploratory hypothesis was that RAPA would improve clinical, functional, inflammatory mediator, and/or cardiovascular outcomes. Data were assessed using the MATLAB^©^ programming environment (version R2023a; The MathWorks Inc, Natick, MA, USA), Excel^©^ (Microsoft Corporation, Redmond, WA, USA), and the GAMLj3 module (Marcello Gallucci) in the jamovi project (2024). *jamovi* (Version 2.6) [Computer Software]. Retrieved from https://www.jamovi.org Longitudinal analyses involving 2–3 time points in the same subject were conducted separately for each outcome by a linear mixed model to evaluate changes from pre-treatment values. Body surface area (BSA) was calculated from patient weight and height using an approximation method developed by Mosteller et al. [42]. Unless otherwise noted, cohort data are presented as mean ± standard deviation and adjusted *p*-values < 0.05 were deemed significant.

For the ELISA data, the primary hypothesis was that RAPA treatment would alter the levels of some inflammatory mediators, so changes between the pre-treatment values and the post-treatment values were assessed for statistical significance using a paired *t*-test in GraphPad Prism. However, given the relatively small sample size in this study (n = 6 for TGF-β, RAGE, and sICAM-1; n = 5 for IL-6), it is recognized that additional testing will be required to validate these findings.

## Results

### Subject and clinical parameters

The study population consisted of 6 male subjects (ages 70–76) who were in good overall health and devoid of any active cardiovascular problems. Their average age was 74.7 ± 2.4 yr. (pre-RAPA). Average subject weight was 83.17 ± 15.5 kg pre-RAPA and 82.4 ± 13.5 kg post-RAPA (p = 0.57). Subject height was 1.74 ± 0.08 m (pre- and post-RAPA). No statistically significant changes occurred in Systolic blood pressure (SBP), diastolic blood pressure (DBP), mean arterial pressure (MAP), or heart rate (HR) (pre- vs post-RAPA). Medications taken by participants are summarized in Table 2 and population parameters are summarized in Table 3.

**Table 2:** Participant medications

Drug class	Number of participants
Dyslipidemia (statins)	5
Dyslipidemia (ezetimibe)	1
Multivitamins	5
ASA (81 mg)	4
Omega 3 Fish Oil	2
Vitamin D	1
Niacin	1
Omeprazole	1
Dilantin	1

**Table 3:** Subject and CMR parameters

	Pre-RAPA	Post-RAPA
Subject Parameters		
Age (years)	74.7 ± 2.4	74.8 ± 2.4
Height (m)	1.74 ± 0.08	
Weight (kg)	83.2 ± 15.5	82.4 ± 13.5
Body Surface Area (m^2^)	2.00 ± 0.20	2.00 ± 0.20
Heart Rate (bpm)	62.2 ± 12.2	65.0 ± 15.4
Systolic Parameters		
ESV (mL)	35.5 ± 6.2	47.1 ± 15.3
SV (mL)	65.6 ± 12.5	80.6 ± 7.7
EF (%)	64.7 ± 5.2	64.1 ± 7.2
CO (L/min)	4.18 ± 1.49	5.25 ± 1.44
CI (L*min^−1*^m^−2^)	2.07 ± 0.61	2.66 ± 0.83
Diastolic Parameters		
EDV (mL)	101.0 ± 15.6	127.7 ± 22.3
pLVFR (mL/s)	254.2 ± 60.4	309.1 ± 62.8
Maximum Acceleration (cm/s^2^)	0.31 ± 0.04	0.35 ± 0.04
Maximum Velocity (cm/s)	52.9 ± 5.0	59.5 ± 8.9
Total Forward Volume (mL)	70.7 ± 14.7	85.5 ± 17.6

All participants completed the study, and none experienced any treatment-related adverse events or complained of any medication side effects. One participant sustained a left wrist fracture due to a fall while roller-skating in an adverse event not related to study participation. All participants were treated with 1 mg/d RAPA, administered orally for 8 weeks, achieving a mean blood concentration of 6.21 ± 1.58 ng/ml after 1 week of treatment which remained statistically unchanged after 8 weeks of treatment (5.94 ± 1.29 ng/ml, *p* = 0.67). These levels correspond to the low therapeutic range for transplant patients (4–15 ng/ml).

Several clinical safety labs manifested statistically significant changes with RAPA administration including declines in WBC, MCV, and MCH and increases in Hgb A1C and LDL. All these tests remained within normal levels and were judged not to be clinically significant. All results of clinical safety tests are summarized in Table 4.

**Table 4:** Changes in clinical safety labs (mean ± std. dev.)

Safety labs	Pre-RAPA	4 wks RAPA	8 wks RAPA
WBC	5.95 ± 1.23	5.53 ± 1.17	5.28 ± 1.13 (*p* = 0.0068)
HGB	15.55 ± 0.87	15.90 ± 0.92	15.63 ± 1.03
HCT	45.65 ± 2.24	46.70 ± 2.59	45.43 ± 2.69
MCV	97.25 ± 4.46	95.78 ± 4.08 (p = 0.019)	94.37 ± 4.03 (*p* = 0.00026)*
MCH	33.12 ± 1.12	32.63 ± 1.20 (p = 0.02)	32.43 ± 1.08 (*p* = 0.0035)*
MCHC	34.07 ± 0.62	34.08 ± 0.79	34.38 ± 0.57
RDW	13.13 ± 0.50	12.63 ± 0.38 (p = 0.029)	12.50 ± 0.34 (*p* = 0.0091)*
Platelets (× 1000)	194 ± 9.74	204 ± 21.36	211 ± 19.54 (*p* = 0.039)
Fasting Glucose	101.33 ± 13.57	108.17 ± 19.19	102.00 ± 17.63
BUN	17.50 ± 2.59	18.00 ± 3.74	15.83 ± 1.83
Creatinine	0.95 ± 0.12	0.93 ± 0.08	0.98 ± 0.22
Sodium	139.17 ± 2.04	139.50 ± 1.76	140.17 ± 1.60
Potassium	3.97 ± 0.20	4.17 ± 0.28	4.05 ± 0.22
Chloride	102.67 ± 2.73	103.5 ± 2.66	104.5 ± 2.07 (*p* = 0.03)
Calcium	9.62 ± 0.26	9.65 ± 0.19	9.38 ± 0.34
Albumin	4.25 ± 0.18	4.33 ± 0.23	4.22 ± 0.26
Total Protein	6.82 ± 0.43	7.03 ± 0.36	6.93 ± 0.36
Alkaline Phosphatase	68.33 ± 21.47	74.67 ± 21.04	75.83 ± 21.71 (*p* = 0.028)
Total Bilirubin	0.90 ± 0.46	0.75 ± 0.35	0.70 ± 0.32 (*p* = 0.045)
AST	20.0 ± 4.15	21.83 ± 5.23	19.5 ± 3.83
ALT	19.67 ± 3.44	19.50 ± 2.95	19.00 ± 1.90
CO2	29.50 ± 2.07	27.0 ± 4.10 (p = 0.032)	27.00 ± 1.67 (*p* = 0.032)
HbA1c	5.48 ± 0.35		5.63 ± 0.37 (*p* = 0.0071)*
Total Cholesterol	158.33 ± 29.86		176.17 ± 31.77
LDL Cholesterol	83.50 ± 23.57		100.50 ± 29.37 (*p* = 0.043)
HDL Cholesterol	56.00 ± 15.52		53.67 ± 12.55
Triglycerides	94.83 ± 40.43		110 ± 34.23
VLDL	18.83 ± 8.09		22.00 ± 6.87
Rapamycin level	6.21 ± 1.41		5.94 ± 1.18

^*^ Consistent with results reported in Kraig, et al. (24)

### Physical performance results

All participants were right hand dominant and completed HG testing although the participant who sustained a left wrist fracture was unable to perform left HG testing at the 8-week time point due to the fracture. All participants completed the 40-foot timed walk with no assistive devices or difficulties. A statistically significant increase (*p* = 0.032, pre-RAPA vs the terminal 8 wk RAPA) in right hand grip strength over the course of the trial was seen. No changes were found in the timed walk. The results of physical function testing are summarized in Table 5.

**Table 5:** Changes in physical function testing (mean ± std. dev.)

Physical function	Pre-RAPA	4 wks RAPA	8 wks RAPA
Hand grip (Right, Kg)	29.39 ± 5.78	26.39 ± 6.54	34.17 ± 4.53 (0.032)
Hand grip (Left, Kg)	30.0 ± 4.53	24.94 ± 6.70	35.73 ± 8.24
40-foot walk (seconds)	8.55 ± 0.53	8.58 ± 0.58	8.24 ± 0.96

### Cardiovascular results

The participants underwent CMR before RAPA treatment began (PRE) and after 8 weeks of daily drug delivery (POST); the period between CMR studies averaged 57.8 ± 2.8 days. By comparing the PRE vs. POST values, each subject served as his own control. Subject weight and therefore BSA, did not change significantly from before to after treatment. CMR data for all 6 subjects were suitable for analysis as described in Methods. Subject heart rates during CMR showed no statistically significant changes (62 ± 12.2 beats/min, PRE; 65 ± 15.4, beats/min POST) throughout the study as did SBP, DBP, and MAP. No significant changes of myocardial mass were observed between PRE and POST time points (*p* = 0.83).

### Systolic cardiac function

**Fig. 1:**
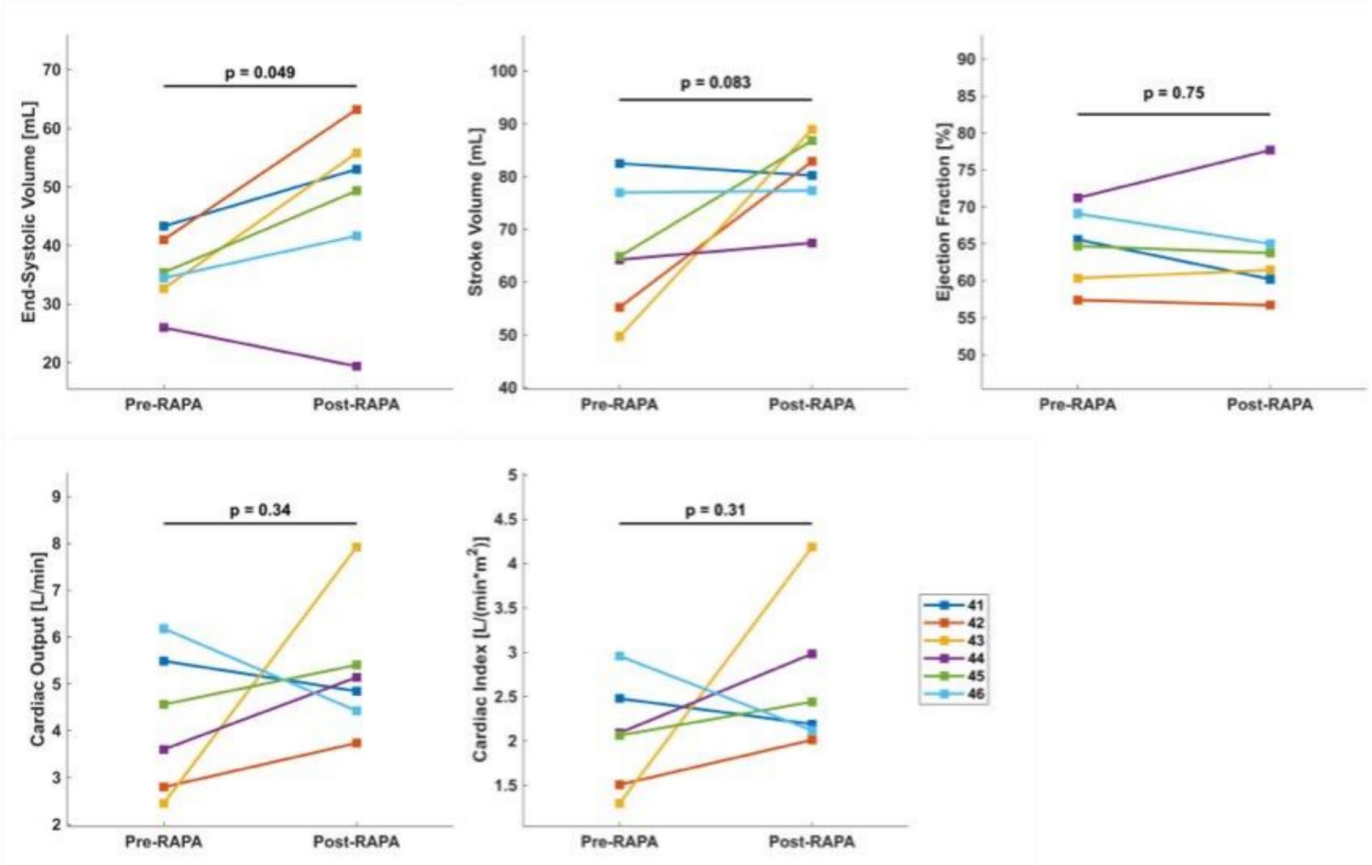
Changes in 5 parameters of cardiac systolic function derived from CMR images. Overall, end-systolic volume showed a statistically significant increase (*p*=0.05) while stroke volume showed a trend toward increasing over the 8 weeks of treatment. Other systolic function parameters showed neither significant changes nor trends. 41 through 46 represent color-coded responses from the 6 participants

All cardiac metrics fit a normal distribution. As illustrated in Fig. 1, measures of systolic function, except for End-Systolic Volume (ESV), did not change significantly between study timepoints, though some parameters showed weak trends. ESV significantly increased after treatment (p = 0.049). All but one subject showed increases in ESV following the RAPA intervention.

Stroke volume (SV) demonstrated a trend toward increasing (*p* = 0.083). No significant changes in ejection fraction (EF) were observed in this study (*p* = 0.75). In most subjects, EF remained relatively the same between study endpoints. Neither cardiac output (CO), nor cardiac index (CI) showed significant changes after treatment (*p* = 0.34 and *p* = 0.31, respectively). Systolic parameters are summarized in Table 3 and Fig. 1.

### Diastolic cardiac function

Parameters of diastolic function were primarily derived from velocity-encoded CMR images acquired on the mitral valve plane. End-diastolic volume (EDV) was the sole parameter derived from volumetric models of cine acquisitions that represented diastolic function. As with parameters of systolic function, all diastolic parameters were normally distributed.

**Fig. 2:**
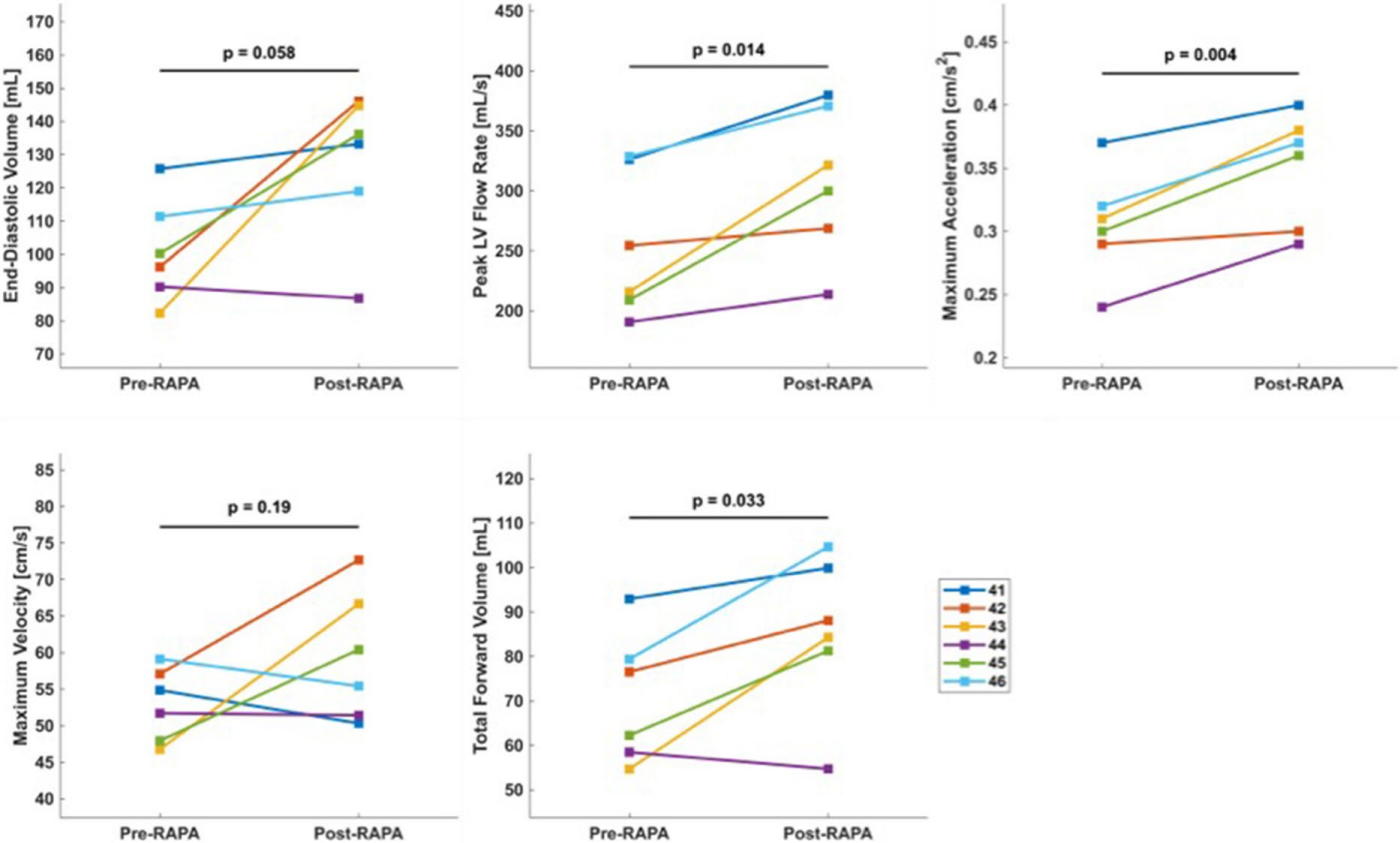
Changes in 5 parameters of cardiac diastolic function from CMR images. Peak leftventricular filling rate (pLVFR), maximum local blood acceleration, and total transmitral forward blood volume demonstrated statistically significant improvements following the RAPA intervention consistent with improved diastolic ventricular function. Changes in end diastolic volume were just above the threshold for significance (*p*=0.058) suggesting a strong trend for improvement. Voxellocalized maximum blood velocity did not show statistically significant improvement; thus, 4 of 5 diastolic function parameters improved with our RAPA intervention. 41 through 46 represent color-coded responses from the 6 participants

As shown in Fig. 2, peak left-ventricular filling rate (pLVFR), maximum local blood acceleration, and total transmitral forward blood volume demonstrated statistically significant improvements following the RAPA intervention. pLVFR demonstrated significant improvement following the RAPA intervention (*p* = 0.014). Furthermore, increases in pLVFR were observed in all subjects. The maximum voxel-localized blood acceleration increased significantly (*p* = 0.004) in all subjects. Additionally, the integrated total transmitral forward blood volume (derived from the flow rate) also showed statistically significant improvement (*p* = 0.033). Overall, these metrics of mitral flow indicate a significant improvement in diastolic function. Changes in EDV were just above the threshold for significance (*p* = 0.058). Voxel-localized maximum blood velocity did not significantly change (*p* = 0.19) but did demonstrate a weak trend.

Despite a small cohort size, several parameters of diastolic function showed improvements with RAPA treatment although HR, SBP, DBP and MAP did not significantly change. No significant or notable interactions between BSA and cardiac measurements were observed. Diastolic parameters are summarized in Table 3 and Fig. 2.

### Endothelial function

**Fig. 3:**
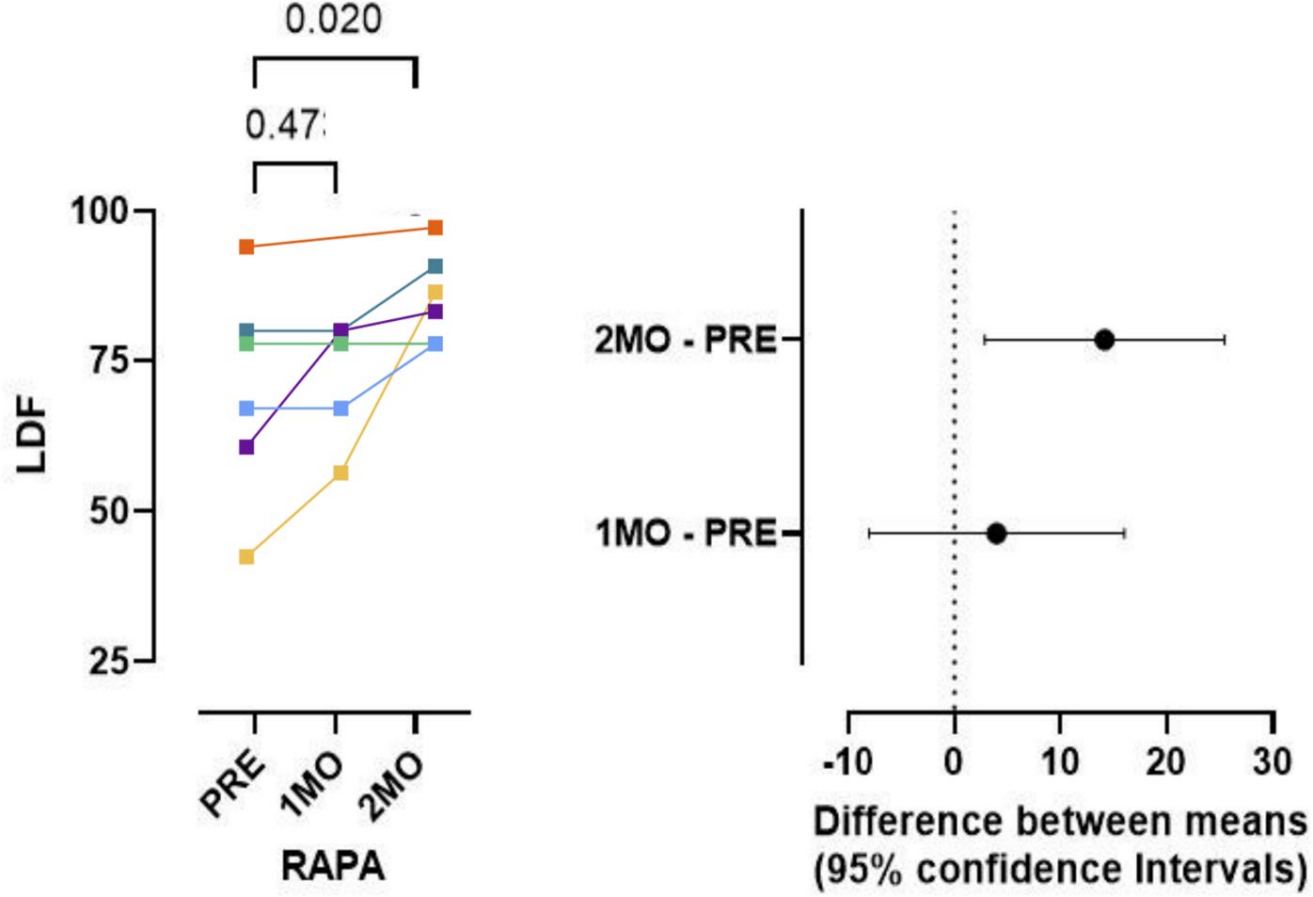
Endothelial function testing by LDF monitoring of the cutaneous vasodilator response to local thermal hyperemia (1). After 5 min with local skin temperature held at 34°C, local skin temperature at the LDF measurement site was rapidly heated to 39°C as an index of NOmediated, endothelium-dependent vasodilation. A statistically significant increase in the local thermal hyperemic response was found after 8 weeks of RAPA treatment which is consistent with improved NO-mediated, endothelium-dependent vasodilation of the cutaneous microvasculature

Microvascular effects of RAPA were tested by assessing local thermal hyperemia in all 6 subjects at 3 time points: before RAPA, after 4 weeks of RAPA, and again after completing 8 weeks of RAPA. There was no significant change in thermal hyperemic responses after 4 weeks of RAPA; however, at this time point, one participant was unable to complete this test due to events unrelated to study participation which reduced the subject number to 5. After 8 weeks of RAPA treatment all 6 participants successfully completed the test; a statistically significant increase in the local thermal hyperemic response was seen (*p* = 0.020) as shown in Fig. 3.

### Inflammatory mediators

As short-term RAPA treatment was shown to improve several parameters of diastolic function and the changes seen were statistically significant even in this small cohort of subjects (n = 6), we posited that associated alterations in the systemic levels of inflammatory mediators might also be detectable. Indeed, in mice, Flynn, et al. [16] had shown that RAPA affected systemic cytokine levels and to a greater extent, within the cardiac tissue. Thus, using banked serum collected from the 6 male subjects in this study before (PRE) and after 8 weeks (POST) RAPA treatment, we performed ELISAs targeting inflammatory mediators that had been previously implicated in cardiovascular health and/or disease, TGF-β [43–45], sICAM-1 [46–48], RAGE [49–51], and IL-6 [52, 53]. These analytes were chosen as they comprise critical components of several inflammatory pathways (reviewed in Zeng, et al. [54]. Moreover, three of these cytokines (TGF-β, sICAM-1, and RAGE, had not been included in our previous published study where multiplex assays were unable to detect a general RAPA-associated decline in pro-inflammatory serum cytokines (Kraig, et al. [24]). Thus, we reasoned that these targets might be more likely involved in the cardiovascular improvements seen.

**Fig. 4:**
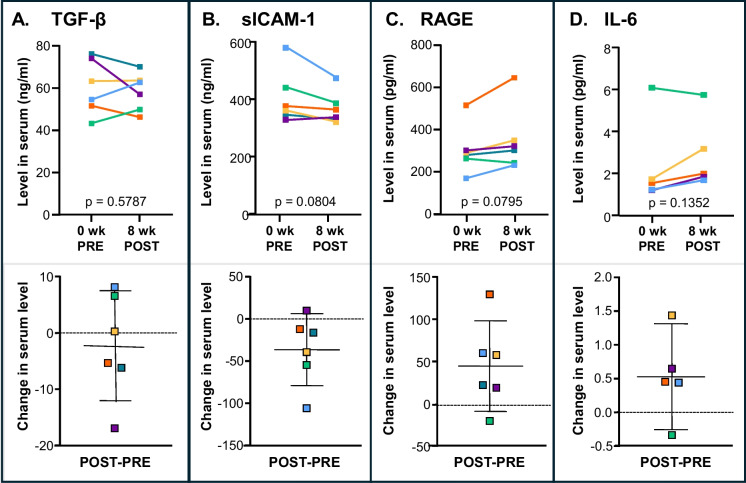
Inflammatory mediator s (pre-treatment to post-treatment). ELISAs were performed on sera collected from study participants at two time points: prior to initiation of the intervention (PRE) and after 8 weeks (POST) of treatment with 1 mg/d oral RAPA. The analytes measured included: Panel **A**: TGF-β, Panel **B**: s(soluble)ICAM-1, Panel **C**: RAGE, and Panel **D**: IL-6. In each case, the top graphs show the actual levels measured at the two time points and the p value is based on the comparison between the two. The bottom graphs show the change in serum concentration (POST-PRE) where values above the dotted lines indicate an increase while points below the dotted line reflect a decreased concentration with treatment; the error bars represent the mean values with a 95% confidence interval. The individual subjects are color-coded as in the previous figures [41–46]. All data are shown with the exception of IL-6 for subject #41; his values for this cytokine were extremely high at both time points and could not be accurately extrapolated. Overall, with this small pilot cohort, no statistically significant change was detected for any of these inflammatory mediators

The ELISA results are shown in Fig. 4 with individual color-coding for the six subjects to allow comparisons across the analyzed cytokines and the cardiovascular outcomes. The top graph for each of the indicated analytes shows the PRE and POST values measured. As expected, there is significant heterogeneity between individuals for the starting values, so we also calculated the change in serum level as POST–PRE (bottom graphs in each panel). Of interest, it was noted that 5/6 subjects showed increased levels of serum RAGE and 5/6 showed decreased sICAM-1. Since RAGE functions as a decoy receptor blocking inflammation, both of these changes could be considered to represent functional improvement with RAPA treatment. However, in neither case was statistical significance met with this small sample size. Thus, the potential regulation of these inflammatory mediators and a potential role in cardiovascular health is worthy of pursuing further in a larger cohort beyond this pilot study.

## Discussion

In our “pilot, proof of concept” study, we found RAPA to be safe and tolerable at a dose of 1 mg daily for 8 weeks in septuagenarian men. In addition, the design of our trial proved to be appropriate to our overall geroscience-based hypothesis as we found statistically significant evidence of RAPA benefits in relatively healthy older men. Specifically, several parameters of diastolic filling of the left ventricle and NO (nitric oxide)-mediated, endothelium-dependent vasodilation were improved by RAPA administration in generally healthy older men; these findings are consistent with preclinical work. That these improvements occurred within a relatively short treatment period of 8 weeks, with a relatively low dose; there were no treatment-related adverse events and no clinically significant changes in safety labs, suggests that a larger clinical trial of similar design can be performed in older persons with the expectation of a reasonable level of safety and tolerability for participants.

Regarding cardiac function effects, statistically significant increases in blood volumes, blood velocities, and blood accelerations across the mitral valve were found post-treatment when compared to pre-treatment baseline levels. In addition, a strong trend toward a statistically significant effect was found for end-diastolic volumes. While no single measure can quantify LV diastolic function [55], as illustrated in Fig. 2, there were significant increases in pLVFR as well as maximum local blood acceleration and volume across the mitral valve after RAPA treatment. Furthermore, LVEDV showed a strong trend toward improvement (*p* = 0.058). Given that there were no changes noted in SBP, DBP, MAP, or HR, it is not likely that undetected changes in afterload confounded our assessment of diastolic function [55].

In contrast to diastolic function, only one significant change in systolic function parameters was observed. ESV increased significantly (*p* = 0.049) and there was also a trend towards increased SV. These findings are like those found in heart transplant patients who receive mTOR inhibitors as part of their immunosuppressive regimen [30, 56–58]. No other systolic function parameters showed significant changes or strong trends that were induced by RAPA. The statistical power of the present pilot study may have been too low to effectively demonstrate significant trends in systolic function parameters.

Overall, prior cardiovascular studies of age-ameliorating effects of RAPA in preclinical models are consistent with those seen in the current study in terms of improved diastolic function and minimal systolic function effects with RAPA treatment. Although multiple mechanisms of aging can be altered by mTOR inhibition, some mechanisms underlying RAPA’s cardiovascular benefits have been defined in preclinical models [59, 60]. Flynn, et al. [16], found that 14 ppm RAPA for 3 months attenuated age-related cardiac hypertrophy and marginally improved systolic function in 24-month-old C57BL/6 mice. In a non-rodent species, RAPA treatment in older dogs significantly improved fractional shortening with associated strong trends toward improved diastolic function and left ventricular ejection fraction (LVEF) though this was not found in a subsequent study using a lower RAPA dose [10, 61]. Both trials were placebo controlled. Dogs in the initial trial received 0.05 mg/kg three times weekly or 0.1 mg/kg three times weekly for 10 weeks and cardiac improvements were noted, whereas in the subsequent trial dogs received 0.025 mg/kg three times weekly for 6 months and were compared to placebo controls but showed no cardiac improvements. These studies indicate that RAPA dosing can greatly influence effects and that lower dosing for longer durations cannot achieve the benefits of greater doses for shorter durations. Specific mechanisms involved in the amelioration of cardiac aging by mTOR inhibition in preclinical species include reduced inflammatory cytokines in blood and even more so in cardiac tissue, attenuated age-dependent protein oxidative damage and ubiquitination, decreased protein turnover rate, and enhanced proteome remodeling [17]. Recently, Chakraborty et al. [62], found that RAPA improved the kinetics of sarcomere shortening and age-associated Ca2 + transient changes compared to age-matched control cardiomyocytes which could have contributed to improved diastolic function in our subjects.

Regarding endothelial function, we found that local thermal hyperemic responses to rapid heating of the skin to 39 °C increased in response to RAPA treatment. We chose the thermal hyperemic approach developed by Choi et al. [1] which has been used in clinical trials with prediabetics and Type 2 diabetics [63]. It is a simple, non-invasive approach to demonstrate altered NO-mediated, endothelium-dependent vasodilation in the skin. Increased NO-mediated, endothelium-dependent vasodilation was observed after systemic RAPA administration for 8 weeks in the present study. This result is consistent with our pilot work that compared cutaneous NO-dependent vasodilation in response to intradermal infusion of the cholinergic agonist methacholine by microdialysis in middle-aged persons [64]. NO-dependent vasodilation was compared between abdominal skin sites treated once daily for 7 days with 8%RAPA ointment versus treatment with the vehicle alone. NO-dependent vasodilation was increased at skin sites treated with 8%RAPA vs control, vehicle-treated sites. Thus, our studies indicate that both systemic and topical RAPA can improve NO-dependent vasodilation in the skin. Our current results demonstrate concomitant improvements in NO-mediated, endothelium-dependent vasodilation and cardiac diastolic function in response to systemic RAPA treatment, suggesting significant potential for cardiovascular function improvements with mTOR inhibitor treatments in humans.

Our endothelial results are similar to those found in the conduit vessels of transplant patients who receive mTOR inhibitors as part of their immunosuppressive regimen and manifest increased flow-mediated vasodilation in the brachial artery [65]. Potential mechanisms for endothelial benefits were elucidated by Lesniewski, et al. [5], who found that RAPA normalized superoxide levels and NADPH oxidase expression while reducing the arterial senescence marker, p19, and increasing NO bioavailability. The exact mechanisms involved in cardiovascular improvements in humans receiving mTOR inhibitors remain to be explored, as well as potential effects on the myriad of pathways dependent on mTOR activity levels [59]. Preliminary analysis of inflammatory mediators known to be important in regulating cardiovascular health suggested that this would be one avenue to pursue in a larger cohort, possibly with longer-term treatment.

### Study limitations

Important limitations of this “proof of concept” pilot study are the small number of subjects (N = 6) and the lack of a placebo control group. While the statistically significant improvements in cardiac diastolic function and in NO-mediated endothelium-dependent vasodilation are not likely due to a placebo effect, we cannot exclude this from our open label trial. Furthermore, while our results showed statistical significance, larger trials of longer duration will be required to examine the clinical significance of RAPA effects on cardiac aging. In addition, longer studies will be needed to ensure RAPA's long-term safety.

Another limitation of our study is that only males were included. Yet, from the earliest preclinical trials of age-amelioration by RAPA, sex differences in efficacy have been reported, including outcomes involving the cardiovascular system [22, 66]. Sex-specific effects of RAPA on cardiac aging clearly require further study. This should be explored as preclinical data indicates that RAPA may exert greater effects in females [22].

## Conclusions

As in our prior trial [24], the present pilot trial shows that 1 mg of RAPA per day is safe and tolerable to healthy older men and that the general design of our trial is all in all appropriate for studying cardiovascular effects of mTOR inhibition by RAPA. Consistent with the geroscience hypothesis, our'proof of concept'pilot clinical trial also demonstrates the potential utility of mTOR inhibitors in ameliorating and perhaps rejuvenating age-related cardiovascular sequelae in healthy older men devoid of clinically detectable cardiovascular disease, particularly for diastolic function and vascular/endothelial function. Given our results, larger and longer clinical trials in generally healthy older men and women are clearly warranted. Furthermore, given that our pilot results demonstrate diastolic and vascular function improvements consistent with pre-clinical findings, the present study strongly supports future clinical trials in patient populations with reduced cardiovascular function, and particularly in disorders of diastolic function such as heart failure with preserved ejection fraction (HFPEF) which is devoid of clear pharmacological interventions.

## Data Availability

Data that support the findings of this study are not openly available due to reasons of sensitivity, but are available from the corresponding author upon receipt of reasonable requests. Data are located in controlled access data storage at UTHSCSA.
